# Incidence of hip replacement among national health insurance enrollees in Taiwan

**DOI:** 10.1186/1749-799X-3-42

**Published:** 2008-09-15

**Authors:** Yu-Shu Lai, Hung-Wen Wei, Cheng-Kung Cheng

**Affiliations:** 1 Institute of Biomedical Engineering, National Yang Ming University, Taipei, Taiwan; 2 Joint Prosthesis Technology Research Center, National Yang Ming University, Taipei, Taiwan

## Abstract

**Background:**

There is no national joint replacement registry in the country of Asia and reports of national outcomes of joint replacement in Asia as yet. Therefore, this study was then to report a national data of the number of hip replacements, incidence rate, demographic characteristics of hip replacement patients, and short-time survival rate after hip replacement of Han Chinese in Taiwan.

**Methods:**

We analyzed 105,688 cases of hip replacements (including primary partial hip replacement, primary total hip replacement and revision of hip replacement) from National Health Insurance research database between 1996 and 2004. The survival rate of primary hip replacement was estimated for each disease by the Kaplan-Meier method.

**Results:**

Average annual number of primary partial hip replacement and primary total hip replacement were 4,257 and 6,206 cases, respectively. The most two common diagnosis of primary partial hip replacement were femoral neck fractures (73.6%, 34% men, mean age 76 years) and avascular necrosis (18.0%, 84% men, mean age 48 years). In primary total hip replacements, the most two common diagnosis were avascular necrosis (46.9%, 79% men, mean age 50 years) and osteoarthrosis (41.6%, 43% men, mean age 60 years). Both the number of primary partial hip replacements and primary total hip replacements increased steadily between 1996 and 2004. The cumulative survival of primary partial hip replacements and primary total hip replacements in all patients were 93.97% and 79.47% in 9 years follow-up, respectively.

**Conclusion:**

Avascular necrosis is the main disease in total hip replacement in Taiwan. The epidemiology of hip diseases was different between Han Chinese (in Taiwan) and Caucasian and the number of hip replacements increased substantially in Taiwan between 1996 and 2004.

## Background

National joint replacement registry is a good solution to record and publish the information for the orthopaedic community on the outcome of joint replacement surgery. Norway (in 1987) [1] and Sweden (in 1979) [2] established national total hip replacement (THR) registry and collected data of arthroplasty from hospitals in the whole country. The main purpose of the registry was to discover inferior results as early as possible in order to avoid inferior implants from being used in large numbers of patients. The statistical analyses reports of registry provided epidemiology, outcomes assessment, and risk factors for revision, etc. However, there is no national joint replacement registry in the country of Asia and reports of national outcomes of joint replacement in Asia as yet. In Taiwan, there is also no official report related to joint replacement like the distribution of diseases between men and women, and survival rate, etc. Therefore, we always rely on the reference of foreign and adopted the medical concept from the Western country. But in clinics, a lot of surgeons realize the patterns of the epidemiology of joint replacement in Taiwan are quite different from the Western. No one could fully understand the distribution of diseases between men and women, and survival rate in whole Taiwan.

Since the implementation of the National Health Insurance (NHI) in Taiwan in 1995, we have accumulated a huge database of clinical cases greater than 96% of the total population in Taiwan. People have received medical health care coverage from this universal national health care system. There were greater than 95% of all the hospitals contained in the NHI databases in Taiwan [3]. Information on all medical treatment undertaken at all medical institutions that contracted with NHI has been recorded in the database since 1996. Therefore, the NHI established a national health insurance research database to respond to current and emerging health issues effectively. The database makes possible the epidemiologic analysis of hip joint disease, because almost all patients who need hip operation treatment are hospitalized to receive hip surgery. Some studies also used data from the NHI in Taiwan [4,5]. The detail information about the NHI program in Taiwan was described in the literature [3].

The purpose of this study was then to report a data of the number of hip replacements, incidence rate, demographic characteristics of hip replacement patients, and short-time survival rate after primary hip replacement in Taiwan.

## Methods

The National Health Insurance program has implemented since March 1995, and the development of the Taiwan's health economy has really taken off. It provides several measures to protect the unemployed, the poor, and the victims of natural and manmade disasters. Moreover, by safeguarding the right of the weak to have access to medical care, the program maintains social order and provides security during this time of economic recession. Until June 2006, there were 22.3 million individuals enrolled in the NHI with a coverage rate of 99% populations (22.7 million) and 18,289 healthcare providers contracted with NHI, representing 91.45% of all providers in Taiwan.

In order to survey the results of hip replacement surgery, we analyzed 105,688 cases of registry for contracted medical facilities and inpatient expenditures by admissions from NHI research database between 1996 and 2004, on the basis of the International Classification of Disease, Ninth Revision, Clinical Modification (ICD-9-CM) code for THR (81.51), partial hip replacement (PHR) (81.52) and revision of hip replacement (including PHR and THR) (81.53) listed as the major operation. These cases included 38,349 cases of primary THR, 55,884 cases of primary PHR and 11,455 cases of revision of hip replacement. We excluded 51 cases (26 cases of primary THR and 25 cases of primary PHR) which aged less than 16 years old. The insurance data was registered by physicians before operation. The data of inpatient expenditures contained six categories including personal information, date of inpatient, diagnosis, operation, expenditures, and hospital information. The items including patient identity, date of operation, birthday, gender, diagnosis, and treatment were used in this study. We compared the patient identity between primary operation (PHR and THR) and revision of hip replacement, and found there were 1,201 and 1,905 cases of revision from the failure of primary PHR and THR originally enrolled in the database, respectively. Survival of primary hip replacement was estimated for each disease by the Kaplan-Meier method [6]. The start date of follow-up was defined as the date of operation. The end-point of survival was defined as the date of revision. For the statistical analyses, we used the software SPSS 10.0 (SPSS Inc. Chicago, Illinois).

## Results

**Figure 1:**
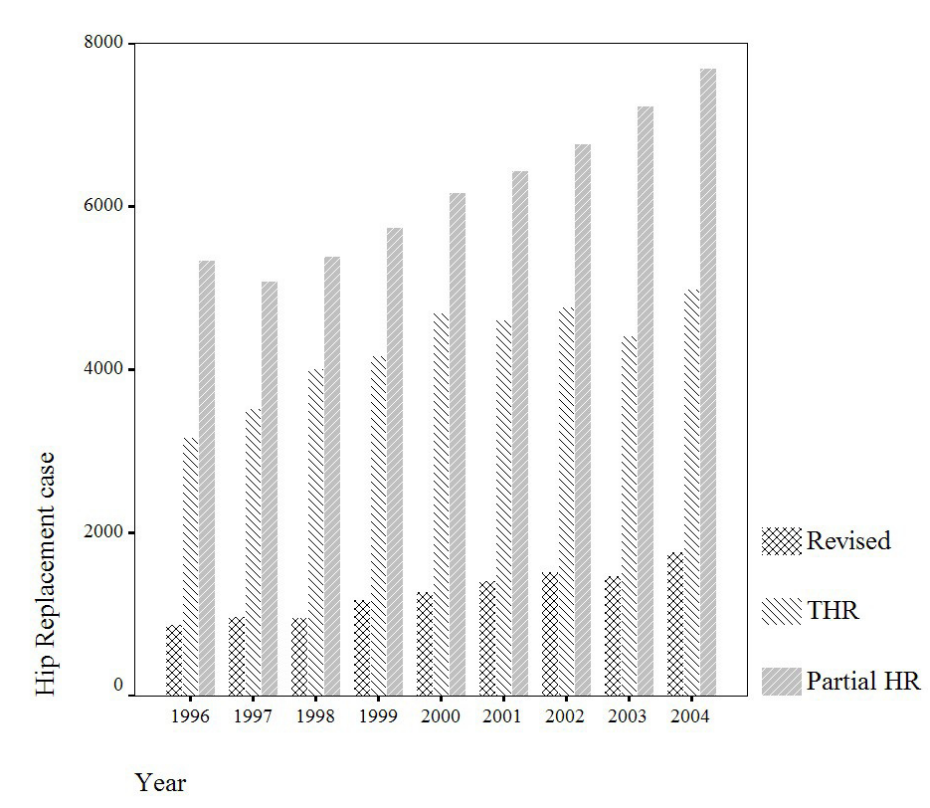
Number of hip replacements recorded in the NHI research database 1996–2004.

Average annual number of primary PHR and THR in Taiwan were 4,257 THRs, 6,206 PHRs, respectively. The incidence rate including primary PHR and THR for male (23 per 100,000) was slightly higher than female (20 per 100,000) per year. The mean age of the patients was 64 years old for the primary PHR and THR, and 49% of the patients were men. The incidence rate (including primary PHR and THR) was increased from 1996 (37 per 100,000) to 2004 (55 per 100,000). Both the trend of primary PHR and THR were increased as shown in Figure 1.

**Figure 2:**
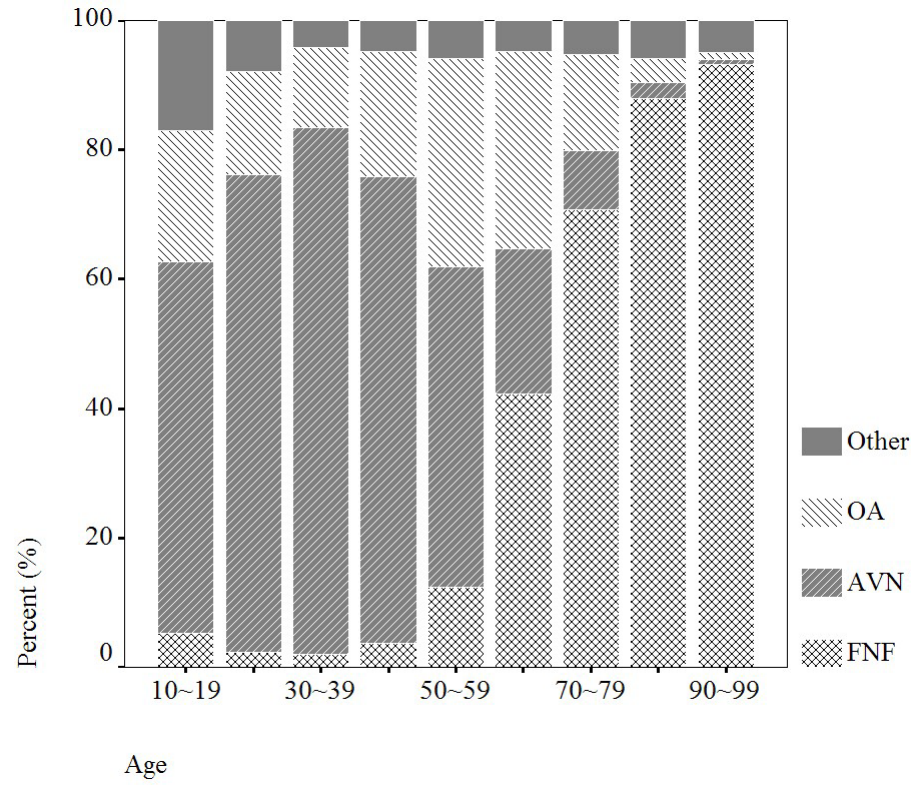
Distribution of diagnosis by age, there were 94,182 observations in 1996–2004.

The most ten common diagnosis in primary PHR and THR were femoral neck fractures (FNF), avascular necrosis (AVN), osteoarthrosis (OA), malunion and nonunion of fracture, rheumatoid arthritis, pathologic fracture, ankylosing spondylitis, congenital anteversion of femur, pyogenic arthritis, and congenital dislocation as shown in Table 1. The main indication for primary PHR and THR in Taiwan was FNF, approximately 42.6% of the index operations were performed due to FNF. Patients of FNF aged less than 60 years were only 4.5%, mean age was 76 years, and 35% were men. Avascular necrosis as indication for primary PHR and THR was 29.8%, age less than 60 years were 76.6%, mean age was 49 years, and 81% were men. Osteoarthrosis as indication for primary PHR and THR was 17.4%, age less than 60 years were 41.6%, mean age was 60 years, and 43% were men. The distribution of diagnosis by age was shown in Figure 2.

**Table 1:** The relationship of gender, age, and percentage of patients to hip disease, in the NHI research database 1996–2004.

	All primary operations including partial and total hip replacement (94,182)			
	
Hip disease	Number (%)	Men (%)	Age < 60 years (%)	Mean age in years
Femoral neck fractures	40143 (42.6)	35	4.5	76
Avascular necrosis	28053 (29.8)	81	76.6	49
Osteoarthrosis	16380 (17.4)	43	41.6	60
Malunion and nonunion of fracture	1273 (1.4)	52	37.2	65
Rheumatoid arthritis	510 (0.5)	21	58	55
Pathologic fracture	370 (0.4)	35	23	68
Congenital anteversion of femur	349 (0.4)	20	68	52
Ankylosing spondylitis	314 (0.3)	93	92	40
Pyogenic arthritis	212 (0.2)	67	65	52
Congenital dislocation	127 (0.1)	16	87	41
Other	6451 (6.8)			

### Partial hip replacements

**Figure 3:**
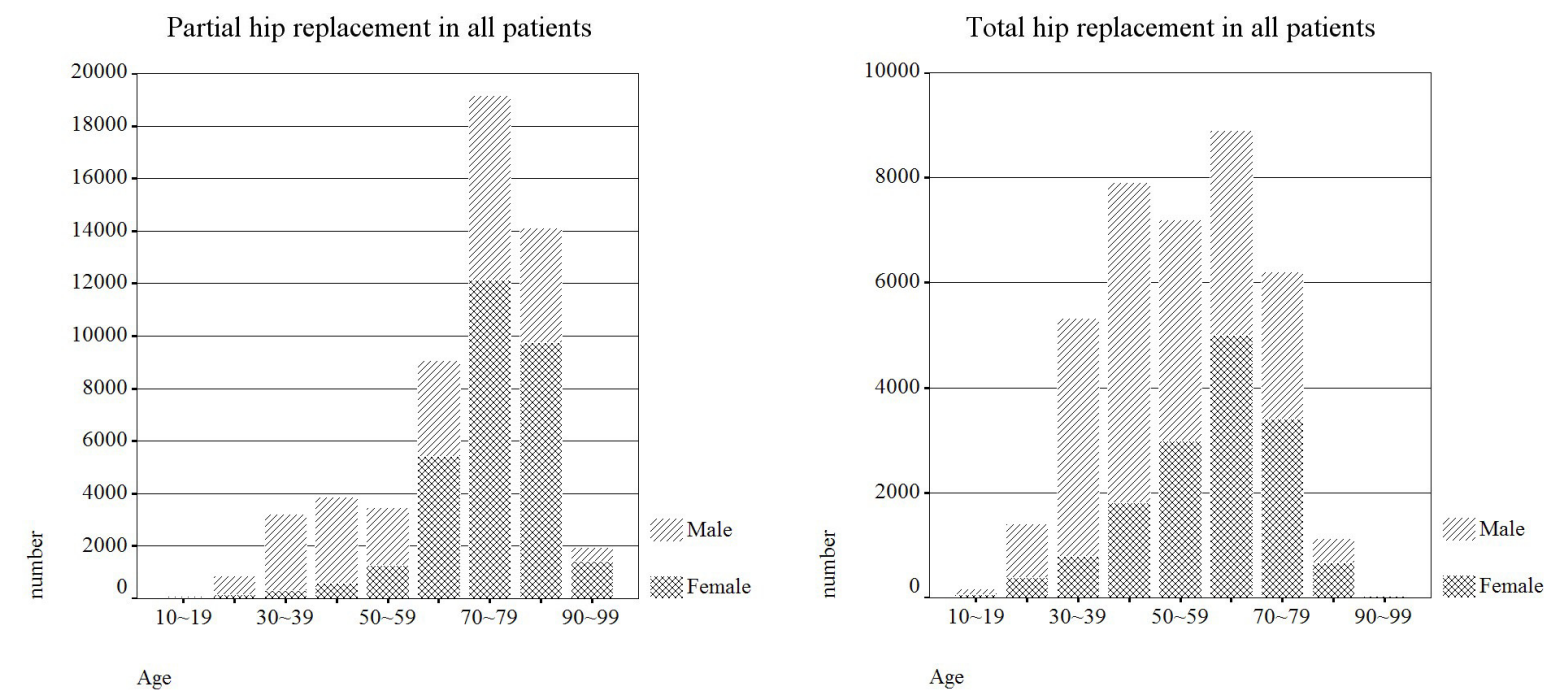
Distribution of age and gender in Taiwan who undertaken (a) primary PHR and (b) primary THR in 1996–2004.

**Figure 4:**
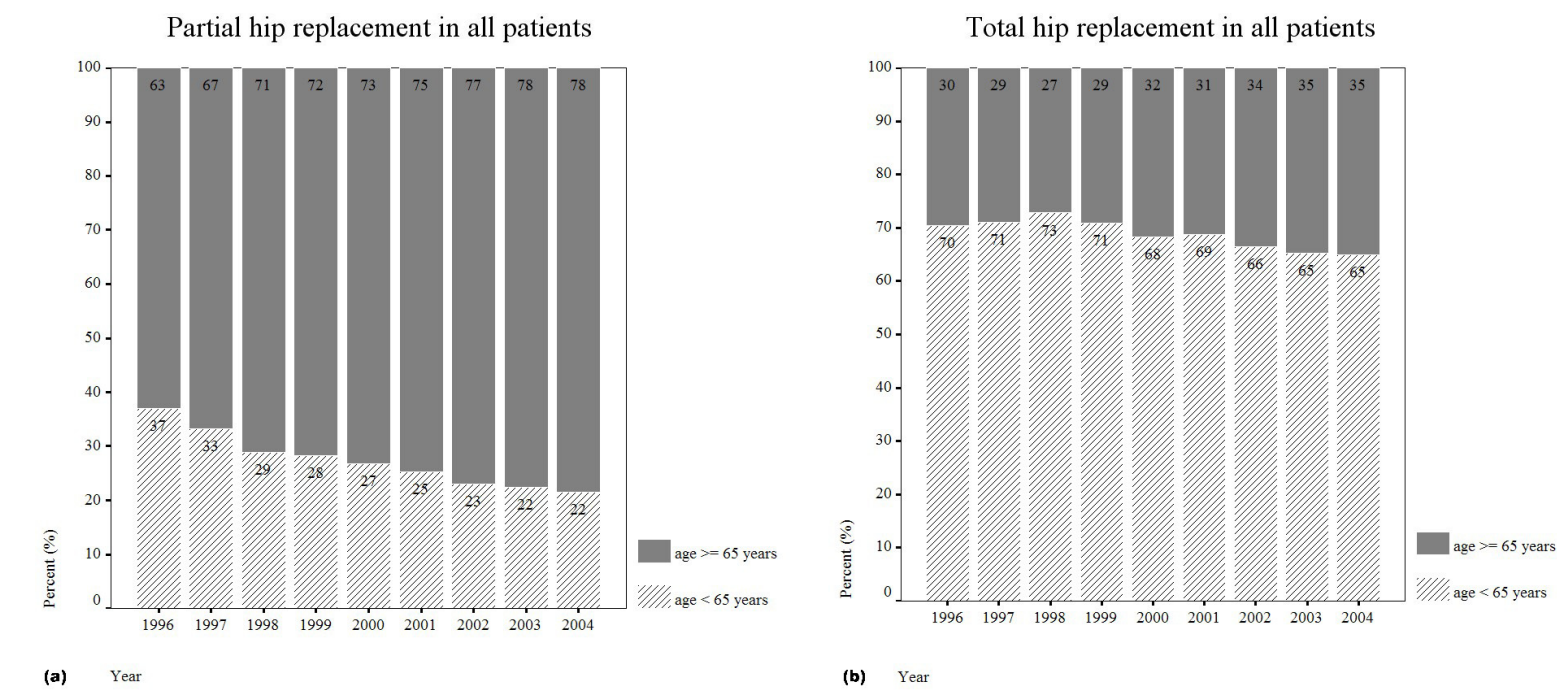
Annual proportion of patients who aged 65 years old (retired on merit) and received (a) primary PHR or (b) primary THR.

The primary PHRs had constituted about 59.3% (n = 55,859) in all primary PHR and THR cases which performed from 1996 to 2004. The annual incidence was 28 per 100,000 inhabitants. The mean age of the patients was 70 years, and 44% were men. The distribution of age and gender in primary PHR was shown in Figure 3a, where the highest incidence age was occurred during 70–79 years. The most three common diagnosis were FNF (70.8%), AVN (18.0%) and OA (0.8%). The relationship of gender, age and percentage of patients to hip disease of primary PHR were shown in Table 2. The annual proportion of patients who aged 65 years (retired on merit) and received primary PHR were increased from 63% to 78% as shown in Figure 4a.

**Table 2:** The relationship of gender, age, and percentage of patients to hip disease of primary partial hip replacement, in the NHI research database 1996–2004.

	All primary partial hip replacement/Revision (55,859/1,201)					
	
Hip disease	Number (%)	**Revision (%)**	Men (%)	Age < 60 years (%)	Mean age in years (range)	**Revision Rate %**
Femoral neck fractures	39557 (70.8)	**577 (48)**	34	4	76	**1.5**
Avascular necrosis	10066 (18.0)	**431 (36)**	84	83	48	**4.3**
Osteoarthrosis	448 (0.8)	**18 (1.5)**	53	49	60	**4.0**
Other	5788 (10.4)	**175 (14.5)**				**3.0**

**Figure 5:**
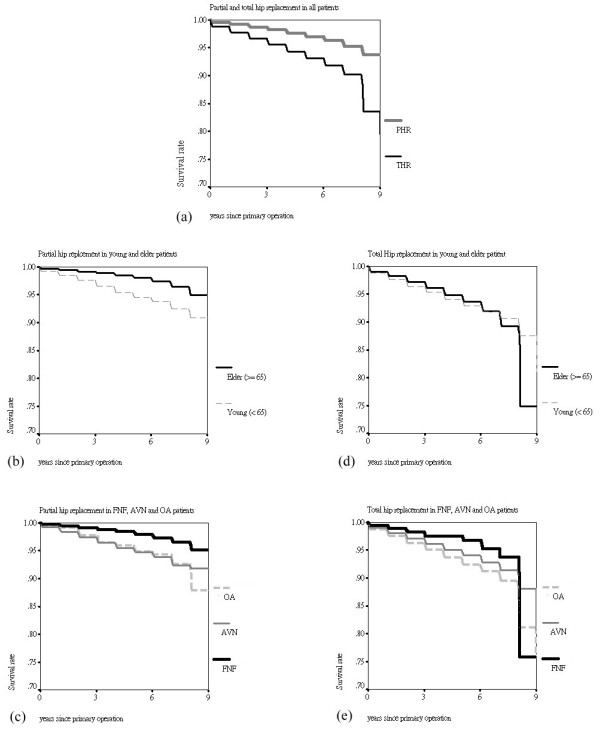
Cumulative survival of (a) primary PHR and THR in all patients, (b) primary PHR in elder and young patients, (c) primary PHR in FNF, AVN and OA patients, (d) primary THR in elder and young patients, and (e) primary THR in FNF, AVN and OA patients.

The relationship between revision rate and diseases was also shown in Table 2. AVN patients had higher proportion (4.3%) to receive revision operation as compared with FNF (1.5%) and OA (4.0%) patients in primary PHR. The main reason for revision was mechanical complication (ICD9-CM 996.4 and 996.5) and the second was infection (ICD9-CM 996.6) in primary PHR (Table 4). The cumulative survival of primary PHR in all patients was 93.97% in 9 years follow-up (Figure 5a). The 9-years cumulative survival rate of the male patients aged above and less than 65 years were 95.02% and 90.39%, respectively (Figure 5b). The cumulative survival of primary PHR in FNF, AVN and OA patients were 95.13%, 91.87% and 87.88% in 9 years follow-up, respectively (Figure 5c).

### Total hip replacements

The primary THRs had constituted about 41.7% in all primary PHR and THR cases which performed from 1996 to 2004. The annual incidence was 15 per 100,000 inhabitants. The mean age of the patients was 55 years, and 60% were men. The distributions of age and gender in primary THR were shown in Figure 3b, where the highest incidence age was occurred during 60–69 years. The most three common diagnosis were AVN (46.9%), OA (41.6%) and FNF (1.5%). The relationship of gender, age, and proportion of patients to hip disease of primary THR were shown in Table 3. The annual percentage of patients who aged 65 years (retired on merit) and received primary THR were increased from 30% to 35% as shown in Figure 4b.

**Table 3:** The relationship of gender, age, and percentage of patients to hip disease of primary total hip replacement, in the NHI research database 1996–2004.

	All primary total hip replacement/Revision (38,323/1,905)					
	
Hip disease	Number (%)	**Revision (%)**	Men (%)	Age < 60 years (%)	Mean age in years (range)	**Revision Rate %**
Femoral neck fractures	586 (1.5)	**22 (1.2)**	48	18	70	**3.8**
Avascular necrosis	17987 (46.9)	**765 (40.2)**	79	73	50	**4.3**
Osteoarthrosis	15932 (41.6)	**858 (45.0)**	43	41	60	**5.4**
Other	3818 (10.0)	**260 (13.6)**				**6.8**

The OA patients had higher proportion (5.4%) to receive revision operation as compared with FNF (3.8%) and AVN (4.3%) patients in primary THR as shown in Table 3. The main reason for revision was mechanical complication and the second was other complications (ICD9-CM 996.7) in primary THR (Table 4). The cumulative survival of primary THR in all patients was 79.47% in 9 years follow-up (Figure 5a). The 9-years cumulative survival rate of male patients aged above and less than 65 years were 74.88% and 81.04%, respectively (Figure 5d). The cumulative survival of primary THR in FNF, AVN and OA patients were 75.77%, 80.68% and 76.94% in 9 years follow-up, respectively (Figure 5e).

**Table 4:** The relationship of gender, and percentage of patients to revision reason of primary partial and total hip replacement, in the NHI research database 1996–2004.

	Revision of PHR(1,201)	Revision of THR (1,905)
Revision Reason (ICD-9 Code)	Number (%)	Number (%)
Mechanical complication (996.4, 996.5)	752 (62.6)	1204 (63.2)
Other complications (996.7)	90 (7.5)	217 (11.4)
Infection (996.6)	102 (8.5)	138 (7.2)
Other	295 (21.4)	346 (18.2)

## Discussion

The major limitation of this study is that the information of the replacement side, implant type and size, surgical approach and cement brand are not recorded in the national health insurance research database. The survival of hip prosthesis was not available. It is the important factor that influences the survival rate of hip replacement. Another limitation is that some cases were failed from severe infection and did not receive revision of hip replacement. Therefore, our results of survival rate would be higher than the true condition.

Our results indicated that the number of primary PHR and THR increased substantially in Taiwan between 1996 and 2004. The trend was pronounced in primary PHR as compared with primary THR (Figure 1). There is a strong relationship between ageing society and the risk of FNF. The age structures indicated that 10.2% of the populations are 65 years old or older in Taiwan at present. The distribution of diagnosis in all primary PHR and THR by age showed that most of the patients (95.4%) with FNF were above 60 years old (Table 1). Aging coincided with a loss of muscle strength, flexibility and balance. Fall down may be the factor caused the FNF in elder patients [7]. In this study, there were 98.5% of FNF patients (39,557) who underwent primary PHR and only 1.5% performed primary THR. This is because that the hemiarthroplasty is recommended for the old patient, who may be occasionally active outside of household [8]. Therefore, for lower expenditure, the law of NHIB provided that FNF should be undergone by PHR of Moore hip prosthesis for patients whose age greater than 80 years old. The NHI also provides the pay of hip arthroplasty for the patients who implant with Moore hip prosthesis. Those policies also explained our results that the annual proportion of patients who aged 65 years old (retired on merit) and received primary PHR were highly and increased year by year as compared with the patients who received primary THR (Figure 4).

In the two groups of primary THR and PHR, we found that the cumulative survival of primary PHR (93.97%) in all patients was higher than primary THR (79.47%). Compared the cumulative survival of primary PHR with Australian (54.6% at 5-years, primary bipolar) [9], our results showed an extra high cumulative survival at 9 years. This may be due to the reason that the patients died before receiving revision replacement because of the old age in Taiwan. The highest incidence of primary PHR and THR were occurred in the age during 70–79 and 60–69 years, respectively (Figure 3a and 3b). The possible reason for lower survival rate in primary THR was that the young patients had more high activity than elder, and it increased the risk of revision. However, the lower survival for younger patients could be due to different implant types, some specific hospitals or regions, some specific diagnosis, or other confounders.

Although the FNF was the main indication for primary PHR in Taiwan (70.8%), the proportion of FNF in primary PHR was low as compared with Australian (94.7%) [9]. Because there were 36% of AVN patients who underwent primary PHR, and the proportion of young patients was higher (76.7%) in AVN group in this study. In the 90's, literatures [10] showed that the hemiarthroplasty was be considered a better way to retained more bone stock for the AVN young patients who had healthy acetabulum. That could be the reason why 36% of AVN patients who underwent primary PHR in this study. However, recent studies showed that hemiarthroplasty is not a good choice for AVN patients and that the acetabulum needs to be done [11].

The reports of primary THR in Taiwan, Norwegian [12,13] and Swedish [2] were shown in Table 5. The AVN (46.9%) was the main indication for primary THR in Taiwan, but in Norwegian and Swedish, primary THR performed due to AVN were lower than 5%. In the United States, the reports of large series of THRs showed that there were about 5 to 12 percent of the procedures were performed for avascular necrosis [14]. The average age of patients with AVN in Taiwan (50 years) is younger than Norwegian (more than 57 years), and the distribution of gender in Taiwan (79% of men) were also different form Norwegian (51% of men) as shown in Table 5. People with a history of alcohol abuse [15] or taking very large amount of cortisone by mouth or injection was predisposed to AVN [12,16]. In Taiwan, physicians found most of the patients used to take some kind of Chinese herbals medicine which contained large amount of cortisone for their arthritis. Therefore, those may be the reasons that the proportion of AVN patients in Taiwan was much higher than Caucasian. From the ethnic point of view, we could estimate the prevalence of hip replacement in China through our results in present study because most people who living in Taiwan are Han Chinese. There are no studies which focus on the prevalence of hip replacement in whole China population. However, It's similar to our results that the clinical studies showed that the most common diagnosis was AVN in Han Chinese as compared with Caucasian [17,18]. The difference between Caucasian and Han Chinese should be considering for a new trend of total hip prosthesis design because of the numerous of AVN patients in Han Chinese. The cumulative survival of primary THR with AVN at 9 years was similar in Norwegian (86%) [13] and Taiwan (80.68%). Other studies showed that the risk of loosening after primary THR is higher in patients with AVN than in those with other diagnoses [19,20]. But our results were similar to the retrospective study of Schneider and Knahr [21] which could not confirm that the AVN is the risk in primary THR as compared with OA.

**Table 5:** The relationship of gender, age, and percentage of patients to hip disease of primary THR, in Taiwan, Norwegian and Swedish.

	Diagnosis	Percentage (%)	Men (%)	Mean age (range) (yrs)
Taiwan (1996–2004)	FNF	1.5	48	70
	**AVN**	**46.9**	**79**	**50**
	OA	41.6	43	60

Norwegian (1987–1999)	FNF	13	21	73
	**AVN**	**0.8**	**51**	**57**
	OA	69	32	70

Swedish (1992–2002)	FNF	11.4	-	76
	**AVN**	**2.9**	**-**	**70**
	OA	74	-	69

It is worth to be taken notice of the difference that the mean age of OA patients in Taiwan (60 years) were younger than Norwegian and Swedish (70 years) as shown in Table 5. Literatures [22,23] reported the much lower incidence of OA among non-whites than among whites but did not point out the difference of age. The cumulative survival of primary THR with OA at 7 to 8 years was similar in Norwegian (90%) [13], Swedish (91%) [2] and Taiwan (89%). Unlike the decrease of cumulative survival steadily in Norwegian and Swedish, it was diving to 76% after 8 years in Taiwan. We could not confirm the reason that it was due to the younger OA patients with a high activity level in Taiwan, because the information of prosthesis is not recorded in the database.

## Conclusion

This study reported statistical data of primary PHR and THR in Taiwan and showed the difference in epidemiology of hip diseases between Han Chinese and Caucasian. AVN is the main disease in primary THR in Taiwan and it is very different form Caucasian in age and gender. Moreover, mechanical complication of hip prosthesis is the main reason for revision. We should be careful with the generalizing results from western countries to other ethnic groups.

## Competing interests

The authors declare that they have no competing interests.

## Authors' contributions

YS wrote the initial manuscript drafts. HW ensured the accuracy of analysis and revised the initial manuscript drafts. YS and HW participated in the design of the study and performed the statistical analysis. YS and CK conceived of the study, and participated in its design and coordination. All authors read and approved the final manuscript.

## References

[B1] Havelin LI (1999). The Norwegian Joint Registry. Bull Hosp Jt Dis.

[B2] Malchau H, Herberts P, Eisler T, Garellick G, Söderman P (2002). The Swedish total hip replacement register. J Bone Joint Surg.

[B3] Cheng TM (2003). Taiwan's new national health insurance program: genesis and experience so far. Health Aff.

[B4] Chang LY, Chang IS, Lu CY, Chiang BL, Lee CY, Chen PJ, Wang JT, Ho HN, Chen DS, Huang LM (2004). Epidemiologic features of Kawasaki disease in Taiwan, 1996–2002. Pediatrics.

[B5] Chien IC, Chou YJ, Lin CH, Bin SH, Chou P (2004). Prevalence of psychiatric disorders among National Health Insurance enrollees in Taiwan. Psychiatric Services.

[B6] Kaplan EL, Meier P (1958). Nonparametric estimation form incomplete observations. J Am Stat Assoc.

[B7] Gale MG, Mitchell HW (1990). Falls in the elderly: part II, balance, strength and flexibility. Arch Phys Med Rehabil.

[B8] Gebhard JS, Amstutz HC, Zinar DM, Dorey FJ (1992). A comparison of total hip arthroplasty and hemiarthroplasty for treatment of acute fracture of the femoral neck. Clin Orthop.

[B9] Australian orthopaedic association (2006). National joint replacement registry annual report.

[B10] Amstutz HC, Grigoris P, Safran MR, Grecula MJ, Campbell PA, Schmalzried TP (1994). Precision-fit surface hemiarthroplasty for femoral head osteonecrosis. J Bone Joint Surg Br.

[B11] Lee SB, Sugano N, Nakata K, Matsui M, Ohzono K (2004). Comparison between bipolar hemiarthroplasty and THA for osteonecrosis of the femoral head. Clin Orthop.

[B12] Abeles M, Urman JD, Rothfield NF (1978). Aseptic necrosis of bone in systemic lupus erythematosus: relationship to corticosteroid therapy. Arch Intern Med.

[B13] Furnes O, Lie SA, Espehaug B, Vollset SE, Engesaeter LB, Havelin LI (2001). Hip disease and the prognosis of total hip replacements. J Bone Joint Surg.

[B14] Mont MA, Hungerford DS (1995). Non-traumatic avascular necrosis of the femoral head. J Bone Joint Surg.

[B15] Jacobs B (1992). Alcoholism-induced bone necrosis. N Y State J Med.

[B16] Cruess RL (1981). Steroid-induced osteonecrosis. J R Coll Surg Edinb.

[B17] Chiu KH, Shen WY, Chan KM (1995). Uncemented porous-coated anatomic total hip replacement in Chinese patients. International Orthopaedics.

[B18] Chiu KY, Ng TP, Tang WM, Yip D (2001). Charnely total hip arthroplasty in Chinese patients less than 40 years old. J Arthroplasty.

[B19] D'Antonio JA, Capello WN, Manley MT, Feinberg J (1997). Hydroxyapatite-coated implants. Total hip arthroplasty in the young patient and patients with avascular necrosis. Clin Orthop.

[B20] Saito S, Saito M, Nishina T, Ohzono K, Ono K (1989). Long-term results of total hip arthroplasty for osteonecrosis of the femoral head. A comparison with osteoarthritis. Clin Orthop.

[B21] Schneider W, Knahr K (2004). Total hip replacement in younger patients: Survival rate after avascular necrosis of the femoral head. Acta Orthop Scand.

[B22] Hoaglund FT, Oishi CS, Gialamas GG (1995). Extreme variations in racial rates of total hip arthroplasty for primary coxarthrosis: a population-based study in San Francisco. Ann Rheum Dis.

[B23] Lau EM, Symmons DP, Croft P (1996). The epidemiology of hip osteoarthritis and rheumatoid arthritis in the Orient. Clin Orthop.

